# Etiology of neonatal seizures and maintenance therapy use: a 10-year retrospective study at Toulouse Children’s hospital

**DOI:** 10.1186/s12887-019-1508-5

**Published:** 2019-04-29

**Authors:** E. Baudou, C. Cances, C. Dimeglio, C. Hachon Lecamus

**Affiliations:** 1 Unit of Pediatric Neurology, Hôpital des Enfants, CHU Toulouse, 330 av de Grande Bretagne-TSA, 31059 Toulouse Cedex, France; 2 Biostatistiques, Informatique Médicale, UMR 1027 Inserm, Université Paul Sabatier, Toulouse, France; 3 Service de Neurologie Pédiatrique, Hôpital des Enfants, CHU Toulouse, 330 avenue de Grande Bretagne-TSA, 31059 Toulouse Cedex, France

**Keywords:** Neonatal seizures, Maintenance therapy, Etiology, Valproic acid, Levetiracetam, Carbamazepine

## Abstract

**Background:**

No guidelines exist concerning the maintenance antiepileptic drug to use after neonatal seizures. Practices vary from one hospital to another. The aim of this study was to investigate etiologies and to report on the use of maintenance antiepileptic therapy in our population of full-term neonates presenting neonatal seizures.

**Methods:**

From January 2004 to October 2014, we retrospectively collected data from all full-term neonates with neonatal seizures admitted to the Children’s Hospital of Toulouse, France.

**Results:**

Two hundred and forty-three neonates were included (59% males, 48% electroencephalographic confirmation). The frequencies of etiologies of neonatal seizures were: hypoxic-ischemic encephalopathy (HIE) (*n* = 91; 37%), ischemic infarction (*n* = 36; 15%), intracranial hemorrhage (*n* = 29; 12%), intracranial infection (*n* = 19; 8%), metabolic or electrolyte disorders (*n* = 9; 3%), inborn errors of metabolism (*n* = 5; 2%), congenital malformations of the central nervous system (*n* = 11; 5%), epileptic syndromes (*n* = 27; 12%) and unknown (*n* = 16; 7%). A maintenance therapy was prescribed in 180 (72%) newborns: valproic acid (*n* = 123), carbamazepine (*n* = 28), levetiracetam (*n* = 17), vigabatrin (*n* = 2), and phenobarbital (*n* = 4). In our cohort, the choice of antiepileptic drug depended mainly on etiology. The average duration of treatment was six months.

**Conclusions:**

In our cohort, valproic acid was the most frequently prescribed maintenance antiepileptic therapy. However, the arrival on the market of new antiepileptic drugs and a better understanding of the physiopathology of genetic encephalopathies is changing our practice.

**Trial registration:**

Retrospectively registered. Patient data were reported to the “Commission Nationale Informatique et Libertés” under the number 2106953.

## Background

Seizures are the most frequent neurological symptom during the neonatal period [1]. The neonatal brain is characterized by a high level of synaptogenesis and neuronal plasticity that explains a physiological hyperexcitability, and thus a vulnerability to seizure [2, 3]. The occurrence is between 1 and 3 per 1000 term newborns [4–7].

Etiologies are mainly symptomatic. They are divided into: vascular (hypoxic-ischemic encephalopathy (HIE), ischemic infarction, intracranial hemorrhage); infectious (intracranial infections); metabolic (metabolic or electrolyte disorder, inborn error of metabolism), and malformation (congenital malformations of the central nervous system). The incidence of epileptic syndromes is less than 10% [8]. On the one hand, benign familial neonatal convulsions (BFNC) and benign idiopathic neonatal convulsions are associated with a favorable outcome. On the other hand, early myoclonic encephalopathy and early infantile epileptic encephalopathy have poor prognoses. The risk of developing epilepsy is about 17.9% according to a meta-analysis [9]. Etiology and electroencephalographic profile are important risk factors. However, designing a universal scoring system capable of providing early prognostic information on epilepsy development seems difficult because of the uncertainty related to etiology and gestational age [10].

The World Health Organization recommends treatment of a crisis lasting more than 3 min or repeated clinical or subclinical crises [11]. There is no strong recommendation about maintenance therapy: indication criteria, drug, or duration. The administration of maintenance therapy should be reserved for newborns at risk of seizure recurrence. This risk is less than 10% when seizure control is achieved and both the neurological examination and electroencephalogram (EEG) are normal. Phenobarbital is the acute treatment of choice in neonatal seizures [12, 13]. However, phenobarbital has been found to increase neuronal apoptosis in newborn rats and to have cognitive side effects in infants [13, 14]. This leads to the prescription of a different antiepileptic drug in maintenance therapy [15]. The duration should be as short as possible [16]. The criteria for discontinuing treatment should be both clinical and electroencephalographic [17].

The aim of this study was twofold: to investigate the incidence of etiologies of neonatal seizures in full-term neonates at the University Children’s Hospital of Toulouse, France and to report our practices concerning the maintenance antiepileptic therapy used.

## Methods

### Study population

This is a retrospective cohort study of consecutive full-term neonates admitted to the University Children’s Hospital of Toulouse from January 2004 to October 2014 with suspicion of seizure during the first 28 days after birth. Neonates whose gestational age was over 37 weeks of amenorrhea with clinical convulsions and an abnormal EEG recording either clinical or subclinical convulsions (spike discharge greater than 10 s) or epileptic abnormalities (spikes, polypoints, wave spikes ...) were included. The exclusion criteria were: prematurity, abnormal non-epileptic movements, normal EEG. Failure to perform an EEG due to an early death of the patient was not an exclusion criterion if the context and the clinical data were highly suggestive of seizures.

### Scheme for neonatal seizure treatment in our center

Emergency treatment is given in case of repeated or prolonged seizures to stop them: as first-line treatment, intravenous (IV) phenobarbital, then IV phenytoin if ineffective. Some patients could have received first-line treatment before admission in our center, and in this case intrarectal diazepam could have been used. Oral maintenance anticonvulsant treatment (valproic acid (VPA)), levetiracetam, carbamazepine ...) is started quickly when there is high risk of seizure recurrence. VPA, then levetiracetam is usually the first-line drug of choice for generalized seizures and carbamazepine in focal seizures. The risk factors for seizure recurrence are: status epilepticus, the need to use several emergency treatments to stop seizures, etiology other than a simple easily correctable hydroelectrolytic disorder, abnormal neurological examination or persistent abnormalities in EEG. A neuropediatric evaluation takes place at 3–4 months with EEG. If the patient has not had a seizure recurrence, the neurological examination is satisfactory, and the EEG does not show any epileptic abnormalities, the treatment is stopped gradually over several weeks. Otherwise it is continued 3 months or more depending on the etiology of convulsions.

### Methods

Neonates diagnosed with seizures were identified through a search in the digital database of the medical information center of the Hospital. Clinical data was extracted from computerized and paper medical records.

Data gathered for each patient included gender, gestational age and place where the first seizure occurred. Neonatal seizures were characterized by type (focal, clonic, subtle, myoclonic, tonic, spasms, tonic-clonic, and infraclinical), as reported in the records, and for newborns presenting several type of seizures, the main type was selected. The delay between birth and first seizure, and the presence of status epilepticus were also reported. Status epilepticus was defined as a convulsion lasting more than 15 min or more than three seizures in 30 min.

The following paraclinical data were collected: EEG reports (seizures recorded, paroxystic events such as spikes), cerebral tomodensitometry reports (TDM), and cerebral MRI reports.

We report the seizure etiology for each patient. The diagnosis of HIE was based on a severe metabolic acidemia (umbilical cord or first neonatal blood sample pH of < 7.0) and/or 5-min Apgar score of < 6 and/or fetal distress (abnormal fetal heart rate or meconium-stained amniotic fluid), associated with a clinical examination corresponding to Sarnat’s score of two or three. Ischemic infarction, cerebral malformations and intracranial hemorrhage, including intraventricular hemorrhage, were diagnosed using neuro-imaging. A diagnosis of bacterial or viral infection required findings of biological inflammatory syndrome in plasma and cerebrospinal fluid or highlighting of the virus or bacterium in the cerebrospinal fluid. The diagnosis of hydroelectrolytic disorders was based on the analysis of a blood sample. Inborn error of metabolism was diagnosed by metabolic tests, with or without genetic confirmation. There is no genetic confirmation of BFNC in our study.

Regarding anti-seizure medication, we distinguished between acute therapy administered intravenously to stop a lasting or repeated crisis and maintenance therapy administered orally to avoid the recurrence of convulsion.

We report the type of drug used and the duration of treatment.

We also report the short and mid-term outcomes (death, epilepsy) and the duration of follow-up.

### Statistics

Study results are presented as numbers and percentages. We performed a comparative analysis between the group of all neonates treated with levetiracetam and a group of neonates treated with valproic acid paired with the gender and type of seizure and adjusted in multivariate analysis of the etiology with the Stata software, version 14.

### Ethics

Patient data were reported to the “Commission Nationale Informatique et Libertés” under the number 2106953.

## Results

### Population

**Fig. 1:**
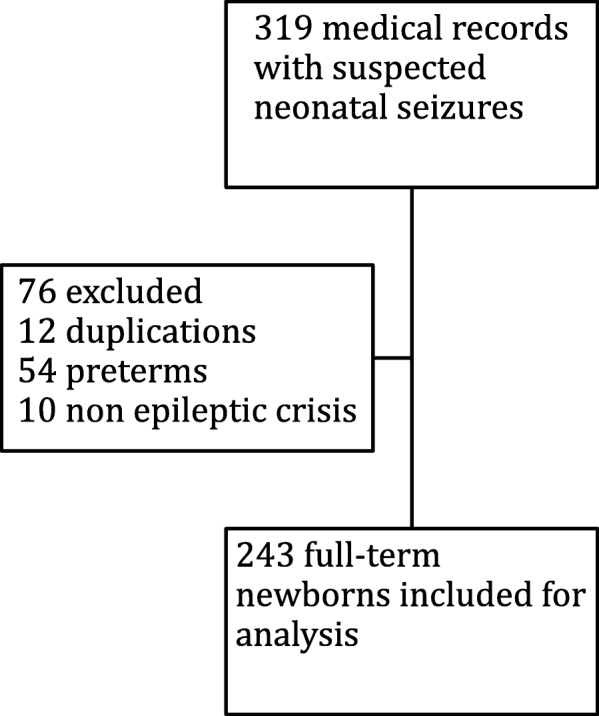
Flow-chart of included newborns

319 full-term newborns with suspected neonatal seizures were admitted to the University Children’s Hospital of Toulouse from January 2004 to October 2014. Of the 243 who were included (Fig. 1), 59% were male and 41% female. The average gestational age was 39 weeks of amenorrhea (range 37–42) and the average birth weight was 3.320 kg (range 1.96–4.8), with 31% births by cesarean section, and 41% spontaneous natural births. The place where the first seizure occurred was known for 213 newborns: 9% at home after maternity unit discharge, 63% in a secondary hospital, and 14% in our center. The initial place of hospitalization was the neonatal intensive care unit (53%), the neonatology unit (43%) and the Department of Neuropediatrics (4%).

### Seizures

Seizures occurred within the first day in 57% of neonates, within 24–72 h in 21%, and after 72 h in 22%. Only 48% had electrographic confirmation of the seizures but 75% had paroxystic events on the EEG. Thirty three percent of neonates presented a status epilepticus. According to the data gathered from patient records, the main type of seizure was focal clonic (35%), followed by multifocal clonic (24%), subtle (20%), myoclonic (2%), tonic (6%), spasms (1%), tonic-clonic (6%), and infraclinical (6%). The mean number of electroencephalograms during hospitalization was 2.23 (range 0–7). Four newborns died before an electroencephalogram was performed and eleven patients’ EEGs showed a “suppression-burst” pattern.

### Etiology

The etiology of neonatal seizures is presented in Table 1. Perinatal asphyxia was the most common cause of seizures in our study group (37%). One patient presented bacterial meningitis, resolved by antibiotic treatment. An inborn error of metabolism was attributed to a patient who presented a severe and prolonged hypoglycemia in the neonatal period and the need of a specific diet in the first months of life, without final diagnosis. Two patients with severe neonatal seizures who developed encephalopathy and pharmacoresistant epilepsy, but with a negative etiologic screening, were classified under severe epileptic syndromes. In 16 patients (6,5%), a diagnosis could not be made based on history, physical examination, laboratory tests, imaging techniques, and metabolic screening tests. Cerebral TDM was performed in 88% of patients. Cerebral MRI was performed later in 65% of patients.

**Table 1:** Etiology of neonatal seizures in term newborns at the University Children’s Hospital of Toulouse from 2004 to 2014

						Death	Epilepsy
Vascular	156 (64%)	HIE	91 (37%)	HIE II	63 (26%)	37 (41%)	7 (13%)
				HIE III	28 (11%)		
		Ischemic infarction	36 (15%)				3 (9%)
		Intracranial hemorrhage	29 (12%)				3 (9%)
Infectious	19 (22%)	Bacterial meningitis	16 (7%)	Streptococcus	11 (5%)	4 (21%)	1 (7%)
				E Coli	4 (2%)		
				Unknown	1 (0,5%)		
		Viral meningoencephalitis	3 (1%)	HSV	2 (1%)		
				Enterovirus	1 (0,5%)		
Metabolic	14 (6%)	Metabolic or electrolytic disorders	9 (4%)	Hypoglycemia	4 (2%)		1 (11%)
				Hypernatremia	3 (1%)		
				Hypocalcemia	2 (1%)		
		Inborn errors of metabolism	5 (2%)	Citrullinemia	1 (0,5%)	4 (57%)	2 (67%)
				Peroxisomal disease	3 (1%)		
				Unknown	1 (0,5%)		
Malformation	11 (5%)	TORCH syndromes	2 (1%)			4 (33%)	6 (75%)
		Neurocutaneous syndromes	2 (1%)	Sturge-Weber syndrome	1 (0,5%)		
				Tuberous sclerosis	1 (0,5%)		
		Gyration abnormalities	7 (3%)				
Epileptic Syndromes	27 (11%)	Benign	17 (7%)	Benign familial neonatal convulsions	7 (3%)		1 (6%)
				Benign idiopathic neonatal convulsions	10 (4%)		
		Severe	10 (4%)	Early myoclonic encephalopathy	2 (1%)	4 (40%)	6 (100%)
				Early infantile epileptic encephalopathy	6 (3%)		
				Others	2 (1%)		
Unknown	16 (7%)						1 (8%)
Total	243					53 (22%)	31 (16%)

*HIE* hypoxic-ischemic encephalopathy, *E coli Escherichia coli*, *HSV* Herpes Simplex Virus, *TORCH* Toxoplasmosis, Other Agents, Rubella, Cytomegalovirus, and Herpes simplex

### Outcomes

The median age of follow-up was 18 months (range 1 month -11 years) for patients having survived the neonatal period.

Fifty-three patients died (22%): thirty-seven HIE, four intracranial infections, four inborn errors of metabolism, four severe epileptic syndromes and four cerebral malformations. Forty- five deaths occurred during the first month of life and eight before the third year of life.

The incidence of epilepsy among patients was 15%. The onset was neonatal in 17 patients. In the other cases, the median age of onset was 10 months. In our cohort, 100% of patients with severe epileptic syndromes, 80% with inborn errors of metabolism, and 75% with cerebral malformations developed epilepsy.

### Acute anti-seizure medication

One third of our cohort received one drug, one third two drugs, and one third, three or more. Phenobarbital was administered to 199 patients (82%), diazepam to 111 (46%) and phenytoin to 79 (32%). Vitamin therapy was tried on only ten patients without success. 86% of newborns treated with diazepam needed a second-line treatment that was phenobarbital in most cases, versus 43% of newborns treated with phenobarbital.

### Maintenance therapy

**Fig. 2:**
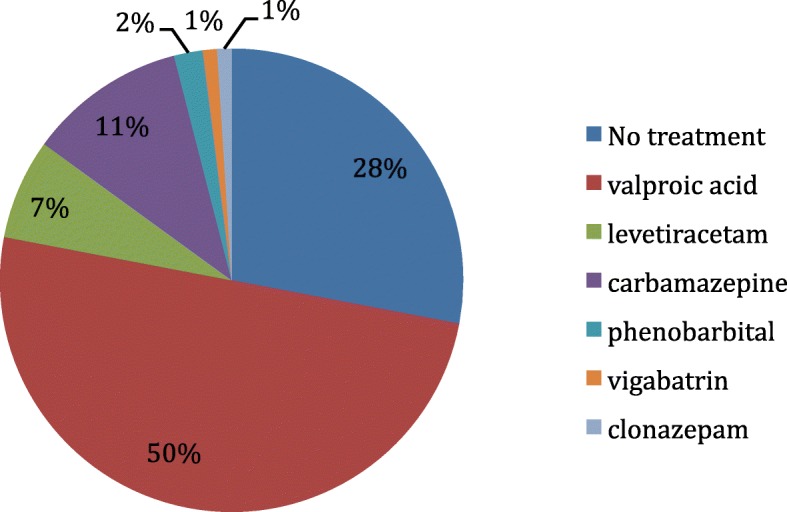
Anticonvulsivant maintenance therapy used in 243 term newborns with neonatal seizures at the University Children’s Hospital of Toulouse from 2004 to 2014

Only 27% of the patients did not receive maintenance therapy: half of them died during hospitalization, and the other half had a few seizures quickly controlled by a single administration of an acute treatment, followed by normal clinical and electroencephalographic post-seizure examinations. In all, 180 patients (72%) were discharged with a maintenance therapy. The therapy used is reported in Fig. 2.

**Fig. 3:**
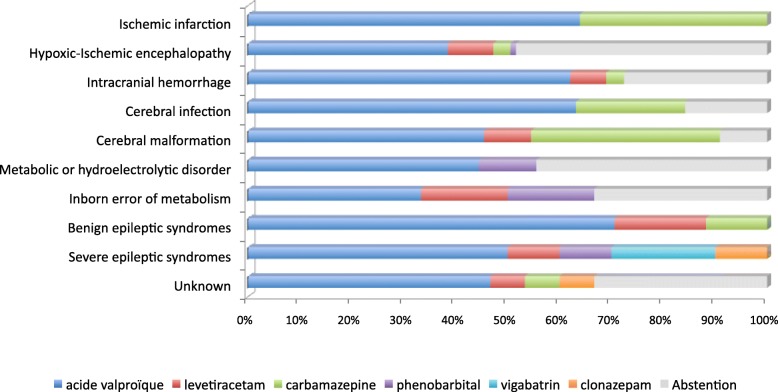
Anticonvulsivant maintenance therapy depending in 243 term newborns at the University Children’s Hospital of Toulouse from 2004 to 2014

In our cohort, the choice of treatment depended mainly on the etiology (Fig. 3). Strokes and severe epileptic syndromes always benefited from a long-term treatment. Strokes, infections and malformations, which usually caused partial seizures, were treated with valproic acid or carbamazepine. Phenobarbital, vigabatrin and clonazepam were used for the difficult cases of drug resistant seizures. Carbamazepine and levetiracetam have been used since 2010.

The mean duration of the first maintenance treatment was 5.2 months for patients who did not develop epilepsy, 4.9 months (SD: 1.61) in hypoxic-ischemic encephalopathy, 4.2 months (SD: 1.69) in ischemic infarction, 4.8 months (SD: 2.14) in intracranial hemorrhage, 4.3 months (SD: 2.26) in intracranial infections, 1.71 (SD: 1.45) in metabolic or electrolyte disorder, 8.69 months (SD: 9.84) in congenital malformations of the central nervous system, 8.5 months (SD: 5.6) in benign epileptic syndromes, and 4.06 months (SD: 2.78) in unknown diagnosis. For patients who developed epilepsy, the treatment lasted at least 2 years. All patients having inborn error of metabolism and severe epileptic syndromes developed pharmacoresistant epilepsy and/or died, and the treatment needed to be switched or associated quickly.

We compare seventeen newborns treated with valproic acid matched to the 17 newborns treated with levetiracetam by the gender and main convulsion type variables and adjusted for the etiology variable. In each group there were 59% boys and 41% girls, 47% of clonic seizures, 23% of focal seizures, 29% of subtle seizures, 6% of myoclonic and 12% of subclinical seizures. Bivariate analysis showed that patients treated with valproic acid received significantly higher numbers of acute antiepileptic drugs compared to patients treated with levetiracetam (Table 2). This difference remained statistically significant in multivariate analysis when considering the type of antiepileptic treatment used in acute first-line therapy. Treatments with levetiracetam and valproic acid were introduced on average with a delay from the first crisis of respectively 1.2 days and 2 days. In terms of outcome, with the low frequency of events, the small size of our groups and the differences in etiology between the two groups, no significant results were found.

**Table 2:** Difference between acute anti-seizure medication in the group treated with valproic acid and the group treated with levetiracetam matched by gender and main convulsion type variables

	Valproic acid			Levetiracetam			
	N	n	*%*	N	n	*%*	*p*-value
Etiology	17			17			
Ischemic infarction		3	17.65		7	41.18	
HIE 2		2	11.76		0	0.00	
HIE 3		0	0.00		1	5.88	
Intracranial hemorrhage		4	23.53		2	11.76	
Intracranial infection		3	17.65		0	0.00	
Cerebral malformation		0	0.00		1	5.88	
Metabolic disorder		1	5.88		0	0.00	
Inborn error of metabolism		0	0.00		1	5.88	
Benign epileptic syndromes		3	17.65		3	17.65	
Severe epileptic syndromes		1	5.88		1	5.88	
Unknown		0	0.00		1	5.88	
Number of emergency drugs	17			16			0.012
0		1	5.88		1	6.25	
1		1	5.88		8	50.00	
2		7	41.18		6	37.50	
3		8	47.06		1	6.25	
First-line emergency drug	17			16			0.097
0		1	5.88		1	6.25	
diazepam		9	52.94		3	18.75	
phenobarbital		5	29.41		12	75.00	
phenytoin		1	5.88				
clonazepam		1	5.88				
Second-line emergency drug	15			7			0.700
diazepam		1	6.67				
phenobarbital		9	60.00		3	42.86	
phenytoin		4	26.67		3	42.86	
clonazepam		1	6.67		1	14.29	
Third-line emergency drug	8			1			0.708
phenytoin		7	87.50		1	100.00	
clonazepam		1	12,50				

*HIE* hypoxic-ischemic encephalopathy

### Evolution

**Fig. 4:**
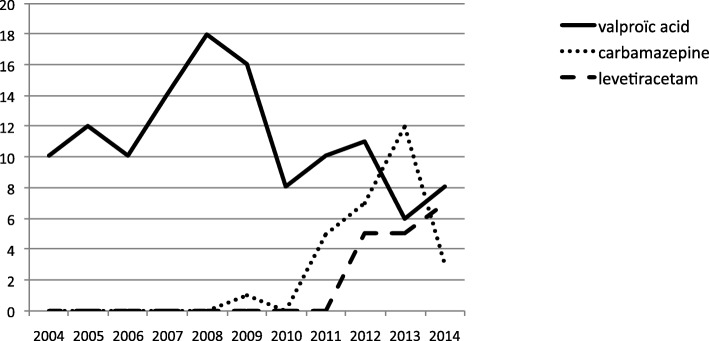
Anticonvulsivant maintenance therapy in neonatal seizures in 243 term newborns at the University Children’s Hospital of Toulouse: Evolution of pratices from 2004 to 2014

Analysis year by year shows a stability of seizures diagnostic rate, use of MRI and duration of maintenance therapy. However, since 2010, new maintenance therapies have been used (Fig. 4).

## Discussion

This study reports the experience of our center concerning the long-term drug management of neonatal seizures depending on etiologies.

The main etiology of full-term neonates admitted to our center because of neonatal seizures was HIE (37%). Compared to a recent American prospective cohort of 426 term and preterm neonates based on EEG diagnosis, the distribution of the various etiologies is similar [18]. In our population, the incidence of each etiology was: 37% (versus 38% in the Glass cohort study) hypoxic-ischemic encephalopathy, 12% (11%) ischemic infarction, 15% (18%) intracranial hemorrhage, 8% (4%) intracranial infections, 3% (4%) metabolic or electrolyte disorder, 2% (3%) inborn errors of metabolism, 5% (4%) congenital malformations of the central nervous system, and 11% (6%) epileptic syndromes. The etiology was unknown in 16 patients. The etiology was not always found in the neonatal period and 7% (9%) of our cohort did not have a diagnosis at the end of the following period.

In this study, the overall incidence of seizures was 0.9 per 1000 full-term live births per year in the Midi-Pyrénées region. In other studies, this incidence was between 1 and 3 per 1000 live births [4–7]. The diagnosis of seizures was based mainly on clinical observation, with a low rate (48%) of electroencephalographic confirmation. Consequently, the incidence of neonatal seizures in our population could have been underestimated. [19] This study highlights the need for our center to improve the use of prolonged EEG video to better diagnose neonatal seizures, especially in newborns at risk.

During neonatal admission, 18.5% of patients died. This is in line with previous publications of Mastrangelo et al. [20] (21%) and Glass et al. (17%) [12]. In our cohort, 15% developed epilepsy. A meta-analysis reported a similar rate of 17.9% in the literature. Etiologies with a poor prognosis were HIE, congenital malformations of the central nervous system, inborn error of metabolism and epileptic syndromes.

Regarding the acute treatment of neonatal seizures, we note that diazepam administered as a first-line treatment usually shows low efficacy. Despite its limited efficacy, diazepam is still used because of the ease of its intra-rectal administration in newborns that still do not have intravenous access. Administration of diazepam should be avoided because it leads to polymedication that will increase the risk of side effects in the neonate and may delay the establishment of an emergency venous route for the administration of phenobarbital.

A maintenance therapy was prescribed for 180 newborns (72%). In a previous study, Bartha et al. [21] reported a rate of 75% of newborns discharged with an antiepileptic therapy. The type of drug used, and duration were not reported in this study. Factors that determined the use of a maintenance therapy were abnormal EEG, neuro-imaging and second-line or further acute antiepileptic treatment in Bartha’s study.

Valproic acid was commonly used in our cohort whatever the etiology of seizures. While other centers use phenobarbital in maintenance treatment, the choice to use other molecules (valproic acid, carbamazepine ...) has been done in our center to avoid neurodevelopmentally related adverse effects related to long-term use of phenobarbital [15]. However, the use of valproic acid does not seem completely safe since serious adverse effects such as hyperammonemic encephalopathy are reported in neonates free of any metabolic disease, apart from overdose [22]. Since 2010, we have used new antiepileptic drugs such as carbamazepine and levetiracetam. Carbamazepine [23, 24] has been used in partial seizures due to stroke, infection, or malformation. Levetiracetam [25–30] has been used in other types of seizures, especially when valproic acid [31–34] was contraindicated, i.e. when liver enzymes were disturbed or when a metabolic disease was suspected. Although, levetiracetam is more and more used, we recall that there is still no marketing authorization for this drug in newborns. Phenobarbital has not been used since 2009 because of its potential cognitive side effects in infants and newborns and the difficulty of finding the correct dose (seizure-free without drowsiness). Vigabatrin has rarely been used in our practice because of the incidence of visual side effects [35–39]. Because of those ophthalmologic side effects, it was only indicated when seizures were difficult to control. [40, 41] Clonazepam was used in similar indications. The small numbers of patients receiving levetiracetam, carbamazepine, vigabatrin, phenobarbital or clonazepam does not allow us to make statistical comparisons about the efficacy and the tolerance of different anti-epileptic treatments, especially because the etiologies of neonatal seizures are different in this group and it is the most important prognostic factor. The subgroup analysis comparing patients treated with levetiracetam, which is now widely used, with a group of patients treated with valproic acid, did not show any difference in the outcome (death and epilepsy) of the patient, maybe because of low effectives. However, it is interesting to note that the number of anticonvulsants used urgently in the treatment of newborns for whom long-term treatment with levetiracetam is set up is significantly lower. This is all the more important since it is known that the risk of adverse effects increases when several treatments are combined.

Recent studies support a targeted therapeutic approach for genetic epileptic encephalopathies based on the molecular dysfunction. For example, *KCNQ2* and *SCN2A* genes are involved in neonatal epilepsy. Their mutations result in sodium and potassium channel dysfunctions. Carbamazepine, is a sodium channel blocker, that also modulates potassium channels, co-localized at the neuronal membrane. Low dose of carbamazepine are effective in this indication and it could be considered as first-line treatment [42–44]. A better understanding of the physiopathology of neonatal epilepsies will help us to determine the more effective antiepileptic drugs to use.

In our cohort, the treatment was discontinued after a control EEG, on the average after 5 months of treatment. The duration depended on the severity of the initial profile, seizure recurrence, etiology and the presence of paroxysmal elements in the electroencephalogram. French guidelines published in 2016 about the treatment of neonatal cerebral arterial infarction, recommend to stop antiepileptic drug 72 h after the last seizure if the clinical examination and EEG are normal or at discharge if there are moderate abnormalities [45]. Achieving earlier discontinuance of maintenance therapy seems to be a major challenge in our practices to limit side effects of antiepileptic drugs.

This study has some biases. The lack of systematic confirmation of seizures at EEG, due to a low use of aEEG, may have led to the inclusion of patients with abnormal non-epileptic movements. The support in different services with the absence of a common protocol of care leads to a great variability of practices. Finally, the retrospective nature induces some missing data and the size of our cohort did not allow us to evaluate the efficacy and tolerance of the different treatments. However, this study allows us to improve the management of neonatal seizures in our center, with the increase of the use of prolonged video EEG, the cessation of diazepam use in the acute treatment of seizures, and the limitation of the duration of the maintenance therapy. We report pratices that have changed during the study period and since the end of data collection and that can’t be used as current practices now.

## Conclusions

In conclusion, despite the lack of systematic electroencephalographic diagnoses, we report a neonatal cohort of full-term newborns comparable with other studies. Although neonatal seizures are often occasional events and the risk of developing epilepsy is about 15%, depending on the etiology, we frequently used a maintenance antiepileptic treatment. No current guidelines allow us to determine the best choice of drug to use in this indication. In our practice, valproic acid was the most commonly prescribed when liver function is normal and metabolic disease excluded. However, the arrival on the market of new antiepileptic drug, and a better understanding of the physiopathology of genetic encephalopathies is changing our practice. Additional studies are necessary to establish recommendations concerning the long-term management of neonatal seizures according to their etiology.

## Abbreviations

BFNC: Benign familial neonatal convulsions
EEG: Electroencephalogram
HIE: Hypoxic-ischemic encephalopathy
TDM: Tomodensitometry
